# Copy number gains of the putative *CRKL* oncogene in laryngeal squamous cell carcinoma result in strong nuclear expression of the protein and influence cell proliferation and migration

**DOI:** 10.1038/s41598-019-56870-5

**Published:** 2020-01-08

**Authors:** Magdalena Kostrzewska-Poczekaj, Kinga Bednarek, Malgorzata Jarmuz-Szymczak, Magdalena Bodnar, Violeta Filas, Andrzej Marszalek, Anna Bartochowska, Reidar Grenman, Katarzyna Kiwerska, Krzysztof Szyfter, Maciej Giefing

**Affiliations:** 1 Institute of Human Genetics, Polish Academy of Sciences, Poznan, Poland; 2 Department of Hematology and Bone Marrow Transplantation, Poznan University of Medical Sciences, Poznan, Poland; 3 Department of Clinical Pathomorphology, Collegium Medicum in Bydgoszcz, Nicolaus Copernicus University in Torun, Bydgoszcz, Poland; 4 Department of Otolaryngology and Laryngological Oncology, University of Medical Sciences, Poznan, Poland; 5 Department of Oncologic Pathology and Prophylaxis, Poznan University of Medical Sciences & Greater Poland Cancer Center, Poznan, Poland; 6 Department of Otorhinolaryngology, Head and Neck Surgery, Turku University Central Hospital and Turku University, Turku, Finland; 7 Department of Tumor Pathology, Greater Poland Cancer Center, Poznan, Poland

**Keywords:** Cancer genetics, Head and neck cancer

## Abstract

Laryngeal squamous cell carcinoma is a major medical problem worldwide. Although our understanding of genetic changes and their consequences in laryngeal cancer has opened new therapeutic pathways over the years, the diagnostic as well as treatment options still need to be improved. In our previous study, we identified *CRKL* (22q11) as a novel putative oncogene overexpressed and amplified in a subset of LSCC tumors and cell lines. Here we analyze to what extent *CRKL* DNA copy number gains correlate with the higher expression of CRKL protein by performing IHC staining of the respective protein in LSCC cell lines (n = 3) and primary tumors (n = 40). Moreover, the importance of *CRKL* gene in regard to proliferation and motility of LSCC cells was analyzed with the application of RNA interference (siRNA). Beside the physiological cytoplasmic expression, the analysis of LSCC tumor samples revealed also nuclear expression of CRKL protein in 10/40 (25%) cases, of which three (7.5%), presented moderate or strong nuclear expression. Similarly, we observed a shift towards aberrantly strong nuclear abundance of the CRKL protein in LSCC cell lines with gene copy number amplifications. Moreover, siRNA mediated silencing of *CRKL* gene in the cell lines showing its overexpression, significantly reduced proliferation (p < 0.01) as well as cell migration (p < 0.05) rates. Altogether, these results show that the aberrantly strong nuclear localization of CRKL is a seldom but recurrent phenomenon in LSCC resulting from the increased DNA copy number and overexpression of the gene. Moreover, functional analyses suggest that proliferation and migration of the tumor cells depend on *CRKL* expression.

## Introduction

Laryngeal squamous cell carcinoma (LSCC) belongs to the highly heterogeneous group of head and neck squamous cell carcinomas (HNSCC). HNSCC is the sixth most common cancer worldwide affecting annually more than 600,000 new patients with mortality rate of approximately 50%^1–3^. Hitherto, there are only two targeted therapies approved for HNSCC patients. The first, Cetuximab is based on a monoclonal antibody directed against the epidermal growth factor receptor (EGFR) found recurrently amplified in head and neck cancer cells^4^. The second, Pembrolizumab use PD1 (programmed cell death protein 1) inhibition as a therapeutic strategy facilitating lymphocytes to recognize cancer cells^5^. The successful application of both therapies demonstrates that better understanding of cancer related molecular pathways and the delineation of therapy targets can translate into clinical practice.

Detailed analysis of recurrent genomic gains in cancer is a validated approach to identify novel oncogenes. Historically, oncogenes were frequently found in large amplified regions like *CCND1* in 11q13 or *EGFR* in 7p12 in LSCC^6,7^. However, along with the development of high resolution techniques it became possible to identify short copy number alterations that could harbor yet undetected oncogenes.

In our previous study, using array-CGH (ang. *Comparative Genomic Hybridization)* platforms we identified *CRKL* (V-crk avian sarcoma virus CT10 oncogene homolog-like, 22q11) as a novel putative oncogene amplified and overexpressed in a subset of LSCC tumors and cell lines^8^. Importantly, amplifications in 22q21.11 region are associated with decreased overall survival of HNSCC patients^9^. The CRKL protein belongs to the adaptor cell signaling proteins which are classified into two groups based on their function and structure^10^. The first group contains membrane localization domains, that have multiple tyrosine phosphorylation sites to bind downstream signaling proteins. The second group, without membrane localization, comprises adaptor proteins containing the SH2 and SH3 domains known to be involved in multiple signal transduction pathways^11^. CRKL belongs to the second group. The protein complexes formed by CRKL and other protein partners are important for biological processes which are recurrently deregulated in cancer progression, like: migration, cell proliferation, survival and adhesion^12–14^.

In this study, we used siRNA - based *CRKL* silencing to determine its effect on cell viability and motility in LSCC cell lines. Additionally, based on immunohistochemical analyses, we propose an explanation, how the oncogenic potential of *CRKL* is triggered by copy number gains.

## Material and Methods

### LSCC cell lines

Three cell lines (UT-SCC-6A, UT-SCC-11 and UT-SCC-29) established at the University of Turku (Finland) from LSCC samples were used in this study. The characteristics of the original samples used to establish the cell lines is shown in Table 1 and was described previously^15^. The cells were grown in 25-cm^2^ flasks in Dulbecco’s Modified Eagle Medium (Gibco, Thermo Fisher Scientific) supplemented with 10% fetal bovine serum (Biochrom, Polgen) at 37 °C under 5% CO_2_.

**Table 1:** Characteristics of the laryngeal cancer cell lines.

Cell line	Sex	Age [years]	T	N	M	Grade	Type of lesion	Survival time [months]
UT-SCC-6A	F	51	2	1	0	1	rec	31
UT-SCC-11	M	58	1	0	0	2	rec	98
UT-SCC-29	M	82	2	0	0	1	pri	124

pri – primary tumor.

rec – recurrent tumor.

F – female, M – Male.

The tumor stage was determined according to the current TNM classification published by the International Union Against Cancer (IUAC).

### Tumor and control samples

Formalin fixed paraffin embedded (FFPE) sections from 40 patients diagnosed with LSCC and treated at the Department of Otolaryngology, Poznan University of Medical Sciences, Poland were collected. The material consisted of tumor tissue and non-tumor laryngeal epithelium. The Ethical Review Board of K. Marcinkowski Poznan University of Medical Sciences approved the study (number 904/06) and the informed conset was obtained from all patients. The characteristics of the tumor samples is shown in Table 2. Both tumor and control samples were revised and selected by two independent pathologists. In the selected samples, the tumor cells content was estimated as approximately 80%.

**Table 2:** Clinical data of patients and primary tumors.

	patients
total number	40
Sex	
male	36
female	4
**Age**	
range	50–84
mean	62,7
median	61
±SD	7,4
**Tumor extension**	
T1	0
T2	0
T3	13
T4	27
**Nodes**	
N0	17
N1	9
N2	11
N3	3
**Metastasis**	
M0	39
M1	1
**Histological differentiation**	
G1	4
G2	26
G3	6
no data	4

### Preparation of cell-blocks from cell lines for immunohistochemistry

The cell pellets from cell lines (UT-SCC-6A, UT-SCC-11 and UT-SCC-29) were suspended in 10% buffered formalin, centrifuged (2000 g/10 minutes) and incubated for 30 minutes with Dubouscq solution. After centrifugation (2000 g/10 minutes) cells were incubated in 200 μl BSA (ang. *Bovine Serum Albumin*) overnight at 4 °C. Subsequently, cell conglomerates were dehydrated in series of ethyl alcohol (80–99.8%), xylen (I-IV) and embedded in paraffin blocks.

### Immunohistochemical staining (IHC)

Paraffin blocks were cut using manual rotary microtome (AccuCut, Sakura, Torrance, USA) to 3–4 µm paraffin sections. Immunohistochemical staining using rabbit anti-CRKL antibody [Y244] monoclonal antibody (1:50; 16 h 4 °C; ab32018; Abcam, Cambridge, UK) was performed according to the protocol described previously and^16^. The antibody complex was detected using EnVisionFlex Anti-Mouse/Rabbit HRP-Labeled Polymer (Dako, Agilent Technologies) and localized using 3-3′diaminobenzidine (DAB) as chromogen.

The protein expression was analyzed at 20x original objective magnification using the light microscope ECLIPSE E400 (Nikon Instruments Europe, Amsterdam, Netherlands). The level of CRKL protein was evaluated according to modified immunoreactive scale (IRS) described by Remmele and Stegner^17^. In detail, IRS was evaluated as the ratio of the percentage of positive stained cells/area (PP) and the intensity of the color reaction (SI) (IRS = SI × PP) and presented in the 0/+/++/+++ scale (0 no protein, + weak expression; ++ moderate expression; +++ strong expression).

### Transfection of small interfering RNAs (siRNAs)

UT-SCC-6A, UT-SCC-11 and UT-SCC-29 cell lines showing various intensity of nuclear *CRKL* expression were selected for siRNA transfection. Approximately 6 × 10^5^ cells were seeded on 24 well plate and cultured to reach 50% of confluence. The cells were transfected with three unique 27-mer duplexes targeting the *CRKL* gene (*CRKL* siRNA, SR300987A, SR300987B, SR300987C, final concentration: 10–20 nM, Origene) or Trilencer-27 Universal Scrambled Negative Control siRNA Duplex (negative control siRNA SR30004, final concentration: 10–20 nM, Origene) using Lipofectamine^TM^2000, according to the standard protocol (Invitrogen). The efficiency of siRNA transfection was measured using RT-qPCR and Western Blot. siRNA duplex with the highest efficacy in gene silencing (UT-SCC-6A and UT-SCC-29: SR300987A, UT-SCC-11: SR300987C) was selected for further analysis.

### RNA extraction and real-time qPCR

Twenty four hours after transfection, total RNA was isolated from cell lines using previously described method^18^. Next, the reverse transcription with Maxima First Strand cDNA kit (Thermo Fisher Scientific, Waltham, Massachusetts, USA) according to the manufacturer’s procedure was performed.

The primers for RT-qPCR were designed with the use of Beacon Designer™ 7.5 software (PRIMER Biosoft International) and verified with the Primer-BLAST database (http://blast.ncbi.nlm.nih.gov/Blast.cgi) to confirm their specificity. As the reference, *GAPDH* and *ACTB* genes were used. Primer sequences are presented in Table 3. The amplification reaction using SYBR^®^Green I was carried out in a total volume of 20 µl containing: 0.2x SYBR®Green I (Sigma-Aldrich), PCR buffer (50 mM KCl, 10 mM Tris-HCl, pH 8.3), 3.5 mM MgCl_2_, 10 nM fluorescein, 0.2 µM of each primer, 0.2 mM of each dNTPs, 0.5 U JumpStart Taq Polymerase (Sigma-Aldrich) and 1 µl cDNA (undiluted reverse-transcription product derived from 0.5 µg RNA in 20 µl reaction volume).

**Table 3:** Characteristics of cDNA primer sequences and reaction conditions.

Gene	Primers sequences	Amplicon length (bp)	Annealing Tm (°C)	Efficiency
***CRKL***	F: 5′ TCAACCTCAGACCACAACTC 3′	166	56	0.91
NM_005207	R: 5′ GATGTCACCAAC—CTCTAATGC 3′			
***ACTB***	F: 5′ CACCACACCTTCTACAATG 3′	162	60	1.00
NM_001101	R: 5′ TAGCACAGCCTGGATAG 3′			
***GAPDH***	F: 5′ GTCGG—AGTCAACGGATT 3′	220	60	0.98
NM_001256799	R: 5′ CCTGGAAGATGGTGATGG 3′			

^*^The hyphen in the primer sequence denotes the exon/exon boundary.

The reactions were cycled 40 times in the following conditions: 95 °C for 15 s, 56 °C (for *CRKL*) or 60 °C (for *GAPDH* and *ACTB*) for 10 s and 72 °C for 15 s during which the fluorescence data were collected. The melting curve was generated to verify the specificity of the product by increasing the temperature from 50 to 95 °C in 0.5 °C intervals per 10 s. The lack of PCR products from the non-reverse transcribed RNA control confirmed the absence of genomic DNA contamination. Each experiment was carried out in triplicate.

Quantitative real-time PCR was performed with the iCycler iQ5 (Bio-Rad) and analyzed with iQ5 Optical System Software 2.0 (Bio-Rad) detection system. Calculations were performed using Gene Expression MacroTM 1.10 software.

### Western Blot

Fourty eight hours after transfection cells were lysed in RIPA buffer (150 mM NaCl, 1.0% NP-40 or 0.1% Triton X-100, 0.5% sodium deoxycholate, 0.1% SDS, 50 mM Tris-HCl, pH 8.0) with Protease Inhibitor cocktail (LabEmpire, Poland). Samples were denaturated with 6 × Laemmli buffer with 5 mM DTT and 40 µg of total protein per lane was loaded onto 12% SDS-PAGE gel. After electrophoresis, proteins were transferred on a nitrocellulose membrane and incubated with the primary antibody: anti-CRKL [Y244] (dilution: 1:500, ab32018, Abcam) at 4 °C, overnight. Membranes were washed and incubated with goat anti-rabbit secondary antibody (dilution 1:40000, ab 97051, Abcam). Rabbit anti-alpha Tubulin (dilution 1:1000, PA5-29444, Invitrogen) antibody was used as a loading control. For protein detection, SuperSignal West Pico Chemiluminescent Substrate (Thermo Scientific) was used. The images were scanned and analyzed with the ChemiDoc XRS+ System (BioRad). With the application of “Relative quantity” tool implemented in ChemiDoc XRS+ system software, the relative quantity (RQ) of CRKL protein was established in each sample with Tubulin as a reference. Based on CRKL and Tubulin protein expression, *CRKL* gene silencing was calculated according to the formula:

$${\rm{CRKL}}\,{\rm{gene}}\,{\rm{silencing}}=100 \% -{\rm{CRKL}}\,{\rm{RQ}}({\rm{siRNA}})/{\rm{CRKL}}\,{\rm{RQ}}({\rm{negative}}\,{\rm{control}})\ast 100 \% $$

### WST-8 assay

The changes in cell proliferation and viability were quantified using the colorimetric assay (Cell Counting Kit-8, CCK-8, Sigma) according to the manufacturer’s instruction. UT-SCC-6A and UT-SCC-11 cell lines transfected with *CRKL* siRNA or negative control siRNA were incubated with the WST-8 reagent in 37 °C for one hour. Thereafter, the absorbance of the culture medium was measured at 450 nm and 600 nm using GloMax microplate reader (GloMax Multi Detection System, Promega): 24, 48 and 72 hours after siRNA transfection. The experiment was performed in triplicate.

### Wound healing migration assay

To analyze the impact of *CRKL* silencing on cell migration, the wound healing assay was used. UT-SCC-6A, UT-SCC-11 and UT-SCC-29 cell lines transfected with *CRKL* siRNA or negative control siRNA were harvested to achieve 100% confluent monolayer of the cells. In order to inhibit cell proliferation and to increase the cell migration potential cells were cultured in medium with reduced FBS concentration (5%) for at least 16 hours before introduction of the “wound” (scratching of the cell monolayer). Images were taken twice in a single field: (I) directly after scratching the culture and (II) after 8 hours (UT-SCC-29) or 20 hours (UT-SCC-6A) using Fluorescence Cell History Recorder, JuLI FL (NanoENTek). The differences in the area covered by cells between the first and the second image determined the level of cell migration. Images were analyzed using the open platform ImageJ (Image processing and analysis in Java). The experiment was performed in triplicate.

### Ethical standards

The Ethical Review Board of K. Marcinkowski Poznan University of Medical Sciences approved the study (No. 904/06) and the informed conset was obtained from all patients. All used methods were performed in accordance with the relevant guidelines, regulations and good labolatory practice.

## Results

### CRKL protein expression correlates with the copy number of the gene

**Figure 1:**
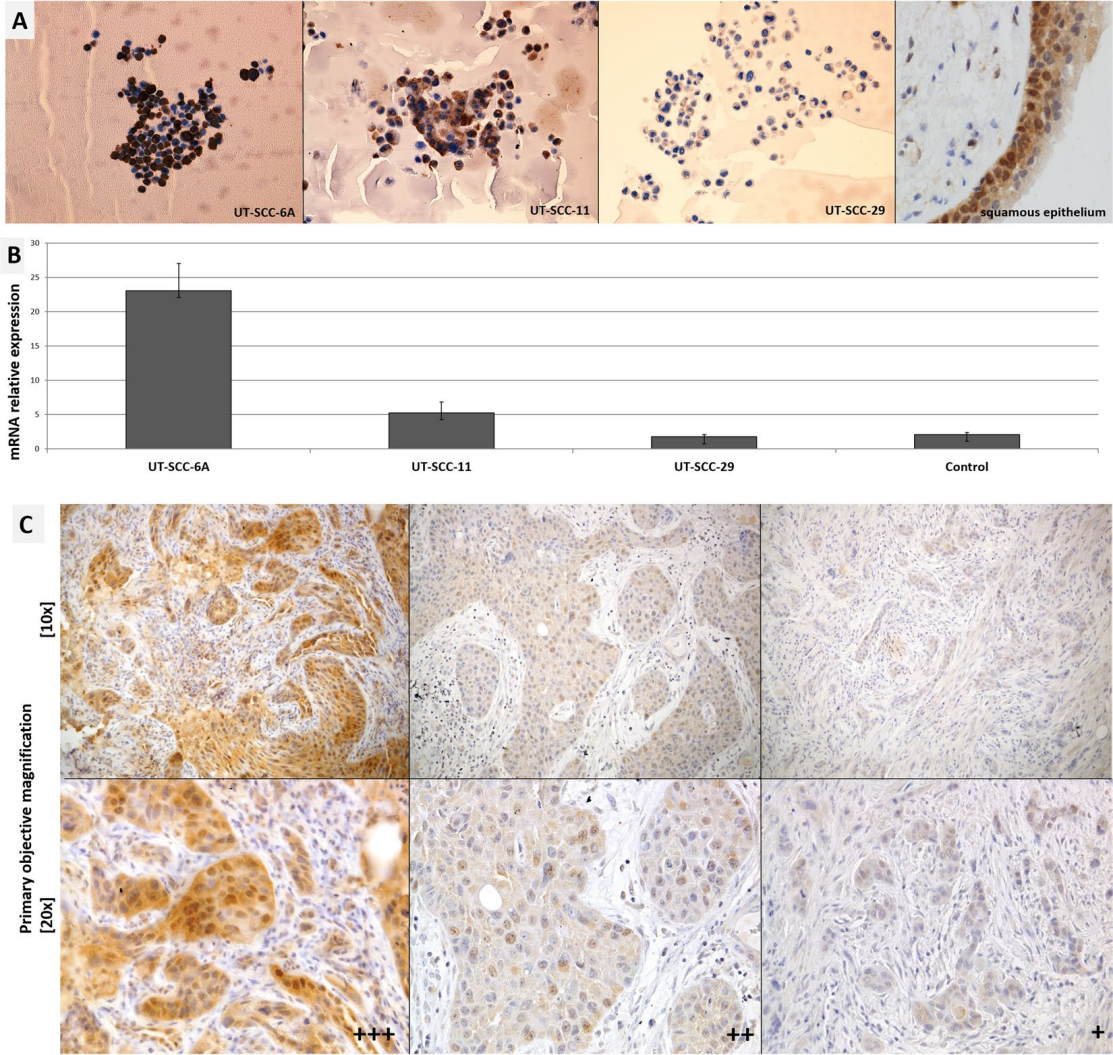
(**A**) Expression of CRKL protein in LSCC cell lines and non-tumor squamous epitelium of the larynx – CRKL present in the basal layer (primary objective magnification 20x). (**B**) *CRKL* mRNA expression shows a good corelation with observed protein expression. (**C**) Examples of CRKL protein expression in LSCC cases. IHC staining of CRKL protein in laryngeal squamous cell carcinoma. The intensity of the expression (+++ strong, ++ moderate, + weak). Nuclei counterstained with hematoxylin show nuclear-cytoplasmic expression.

To establish the intensity and subcellular localization of CRKL protein in laryngeal squamous epithelium under physiological condition we performed immnunohistochemical staining using the non-tumor laryngeal squamous epithelium (Fig. 1A). In this sample, the protein shows moderate nuclear-cytoplasmic expression in the layer of proliferating basal cells but is completely absent in superficial cell layer.

Thereafter, in order to analyse if the copy number alterations of the *CRKL* gene result in differences in the expression of CRKL protein we have performed IHC staining on LSCC cell lines (n = 3) and LSCC tumors (n = 40) (Fig. 1A,C). Consistent with our previous gene copy number and mRNA expression results^8^ the UT-SCC-29 cell line, with diploid copy number for *CRKL*, showed weak cytoplasmic expression of the protein (+). In contrast, the UT-SCC-11 cell line with 13 copies of the gene showed moderate nuclear-cytoplasmic protein expression (++). Last, the UT-SCC-6A cell line with 143 gene copies was found with aberrantly strong nuclear abundance of the CRKL protein (+++). In line, the level of CRKL protein correlated with *CRKL* mRNA expression in the analyzed cell lines (Table 4 and Fig. 1B). These results demonstrate a shift towards nuclear expression of the CRKL protein in cell lines with additional gene copies.

**Table 4:** CRKL immunohistochemicall staining in LSCC.

Cell lines	Intensity of nuclear expression	Expression	Relative DNA copy number^*^
UT-SCC-6A	+++	strong nuclear-cytoplasmic	111 additional copies
UT-SCC-11	++	moderate nuclear-cytoplasmic	11 additional copies
UT-SCC-29	+	weak cytoplasmic	diploid

^*^Based on Kostrzewska-Poczekaj 2010^8^.

In order to identify the frequency of abberantly strong nuclear abundance of CRKL we performed immunohistochemical staining (IHC) of LSCC sections. This analysis revealed the presence of cytoplasmic expression of CRKL protein in all 40/40 (100%) tumor samples. Additional nuclear expression was observed in 10/40 (25%) of the analyzed samples. Detailed analysis of the intensity of the nuclear protein expression demonstrated 7/40 (17,5%) cases with weak nuclear protein expression (+), 2/40 (5%) cases with moderate (++) nuclear expression, and 1/40 (2.5%) case with strong (+++) nuclear abundance of the analyzed protein (Fig. 1C). Taken together these results show that aberrant nuclear localization of CRKL is a seldom but recurrent phenomenon in LSCC.

### LSCC cells show reduced cell proliferation after *CRKL* downregulation

**Figure 2:**
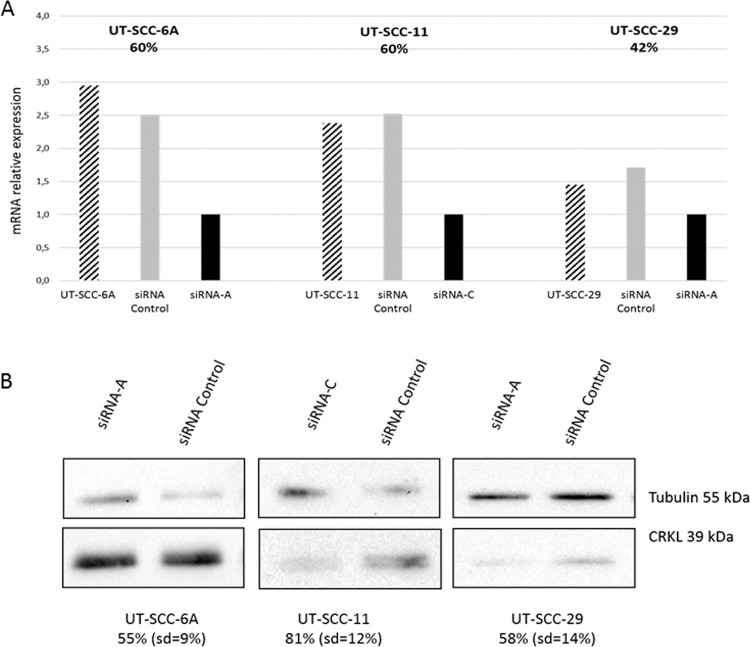
SiRNA mediated *CRKL* knockdown in UT-SCC-6A, UT-SCC-11 and UT-SCC-29 cell lines (RT-qPCR and Western Blot).

**Figure 3:**
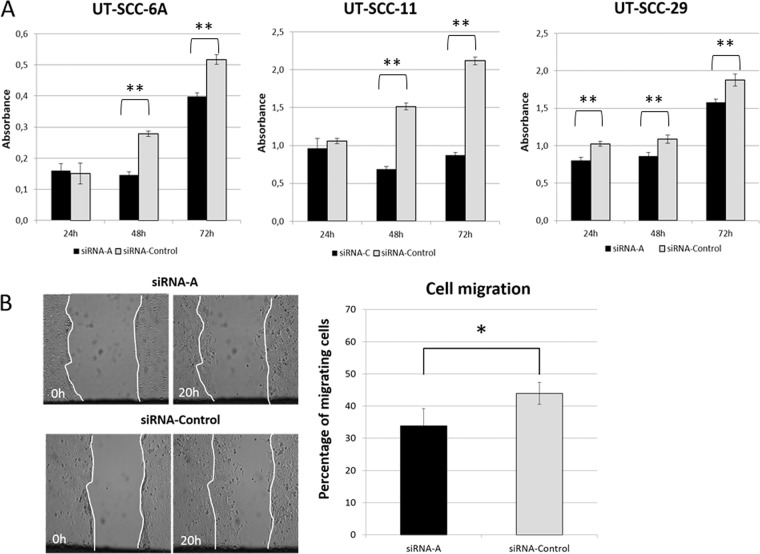
(**A**) *CRKL* knockdown significantly suppressed cell proliferation in UT-SCC-6A, UT-SCC-11 and UT-SCC-29 cell lines. (**B**) *CRKL* gene knockdown significantly suppressed cell migration in UT-SCC-6A cell line. [*statistically significant p < 0.05, **statistically significant p < 0.01].

To analyze the impact of *CRKL* gene on laryngeal cancer cell proliferation, the UT-SCC-6A, UT-SCC-11 and UT-SCC-29 cell lines were transfected with siRNA duplexes targeting *CRKL* or with a negative siRNA as a negative control. Significant loss of *CRKL* mRNA expression and subsequent protein expression was observed in all analyzed LSCC cell lines. In UT-SCC-6A, *CRKL* expression was decreased by 60% on mRNA level and by 55% (SD = 9) on protein level. For UT-SCC-11, the expression was reduced by 60% and 81% (SD = 12) on mRNA and protein level. Whereas, in UT-SCC-29, *CRKL* expression was decreased by 42% on mRNA level and by 58% (SD = 14) on protein level, respectively (Fig. 2, Supplementary Fig. S1). For both cell lines with amplification no changes in cell proliferation as compared to the negative control were observed after 24 hours of incubation. However, after 48 and 72 hours post transfection the proliferation of both *CRKL* siRNA downregulated cell lines was significantly reduced as compared to the negative control. In details in UT-SCC-6A cell line after 48 hours (p = 0,000076) and after 72 hours (p = 0,000724) and in UT-SCC-11 cell line after 48 hours (p = 0,000106) and after 72 hours (p = 0,000047) (Fig. 3A). In the UT-SCC-29 cell line without amplification the significantly reduced proliferation in the siRNA downregulated cells compared to the negative control was already observed after 24 hours (p = 0,002526) as well as after 48 hours (p = 0,012579) and 72 hours (p = 0,003555) (Fig. 3). This collectively demonstrates the importance of *CRKL* gene in the process of cell proliferation and/or viability.

### LSCC cells show reduced cell migration after CRKL downregulation

To analyze the cell migration potential after siRNA mediated silencing of *CRKL* the wound healing assay was performed in three LSCC cell lines. The average migration efficiency of the UT-SCC-6A cells (33.8% of the covered area) after *CRKL* gene knock down was significantly reduced (p = 0.0412) compared to the average migration efficiency of negative control cells (43.9% of the covered area) (Fig. 3B). Interestingly, for the UT-SCC-11 cell line for which the *CRKL* knockdown was more efficient (81%), medium depletion was lethal for the cells transfected with siRNA, while this effect was not observed for the cells transfected with negative control. On the other hand, the average migration efficiency of the UT-SCC-29 cell line (62.9% of the covered area) after *CRKL* gene knock down was not significantly reduced (p = 0.5408) compared to the average migration efficiency of negative control cells (84.8% of the covered area). Althought the tendency to reduce cell migration after decreased *CRKL* gene expression was preserved. The lack of significance may demonstrate diminised dependence of this cell line on CRKL compared to the other cell lines with *CRKL* amplifications. These results together show that *CRKL* contributes to the process of cell migration in LSCC cell lines with *CRKL* amplifications.

## Discussion

The number of molecular findings, which translate into diagnostic approaches or treatment modalities of various malignancies is systematically increasing. In light of this, amplifications that result in overexpression of oncogenes may be successfully used as diagnostic markers and indicate potential therapeutic targets. Our recent study has demonstrated that *CRKL* (22q11) is seldomly but recurrently amplified in laryngeal squamous cell carcinoma^8^. Morover, *CRKL* is known to be overexpressed in a number of different cancer types, including these of breast^19,20^, gastric^21,22^, endometrial carcinoma^23^ and lung^24,25^. Overepression of CRKL is also present in colorectal cancers^26^ and cervical cancer samples^27^. Together these findings suggest that *CRKL* may be a potential target for novel anti-cancer therapies in a subpopulation of patients which show a tumor specific amplification/overexpression of this gene.

Therefore, in this study we intended to further analyze the expression pattern of *CRKL* in LSCC. With the use of IHC staining we have established the localization of CRKL protein in the squamous epithelium of the non-cancer larynx and tumor samples. We have demonstrated significant overexpression of the protein in a subset of LSCC cases but also showed differences in cellular localization of the protein, in regard to *CRKL* DNA copy number of the respective cell lines. In the cell line without *CRKL* amplification (UT-SCC-29) weak cytoplasmic localization of the protein was observed. However, in UT-SCC-6A and UT-SCC-11, where *CRKL* is amplified, cytoplasmic expression was maintained but additionaly a moderate or strong nuclear staining was observed. Importantly, in non-cancer laryngeal epithelium we demonstrated that CRKL shows moderate nuclear-cytoplasmic localization only in the proliferating cells. This points to its putative significant role in the nucleus during cell proliferation. We hypothesize that the increased gene copy number triggers gene overexpression and elevated accumulation of CRKL protein in the nucleus, which in turn enhances cell proliferation and tumor development. Similar findings concerning the sub-celular expression of CRKL protein were previously reported for pancreatic carcinoma and pancreatic ductal carcinoma^28^, suggesting that it might be a general feature, not only relevant to LSCC. Hovewer, no correlation between clinical or histological parameters and the nuclear CRKL protein localization was observed in lung cancer^25^ and gastric cancer^21^.

To further investigate *CRKL* and its potential oncogenic activity in LSCC we performed functional analyses aimed at determining the effect of siRNA knockdown on proliferation and migration of the tumor cells. We show that *CRKL* knockdown leads to statistically significant reduction of cellular proliferation compared to the control cells after transfection. This effect was significant in all analysed cell lines, what suggest an important role of CRKL in proliferation regardles its expression level and localization, but severly more pronounced in the cell lines with *CRKL* overexpression.

Moreover, the importance of *CRKL* in proliferation is shown by the fact that expression of CRKL in normal tissue was observed only in the layer of proliferating basal cells. Therefore, in line with literature reports that link CRKL overexpression with cancer^28,29^, we show that CRKL is crucial for maintaining proliferation of at least a fraction of LSCC cases.

Moreover, in line with previous reports showning that CRKL oncogeneity manifests though the deregulation of cell adhesion what results in changes in cell migration potential in LSCC^14,25,30^ we found that cell migration was significant decreased (p < 0.05) after downregulation of the *CRKL* gene. Inerestingly, this effect was significant only in the cell line with CRKL copy number amplification and overexpression but not in the diploid cell line what suggest a CRKL dependence only in cells harbouring amplifications of the gene.

In summary, our study demonstrate that CRKL is seldomly but recurrently overexpressed in cells with amplification of *CRKL* gene and that it promotes cell proliferation and migration. Therefore we suggest CRKL as a potential therapeutic target for a subgroup of laryngeal cancer patients with additional copies of *CRKL* in the tumor cells.

## Supplementary information

Supplementary Figure S1.
