# Supplementary material for: Copy number gains of the putative CRKL oncogene in laryngeal squamous cell carcinoma result in strong nuclear expression of the protein and influence cell proliferation and migration
Source: Sci Rep. 2020 Jan 8;10:24. doi: 10.1038/s41598-019-56870-5 (PMC6949282; doi:10.1038/s41598-019-56870-5)
Supplement: Supplementary file 1 — Supplementary Figure S1. [file 41598_2019_56870_MOESM1_ESM.docx]

**Copy number gains of the putative *CRKL* oncogene in laryngeal squamous cell carcinoma result in strong nuclear expression of the protein and influence cell proliferation and migration**

Magdalena Kostrzewska-Poczekaj^1^, Kinga Bednarek^1^, Malgorzata Jarmuz-Szymczak^1,2^, Magdalena Bodnar^3,4^, Violeta Filas^5^, Andrzej Marszalek^5^, Anna Bartochowska^4^, Reidar Grenman^6^, Katarzyna Kiwerska^1,7^, Krzysztof Szyfter^1^, Maciej Giefing^1^

1. Institute of Human Genetics, Polish Academy of Sciences, Poznan, Poland

2. Department of Hematology and Bone Marrow Transplantation, Poznan University of Medical Sciences, Poznan, Poland.

3. Department of Clinical Pathomorphology, Collegium Medicum in Bydgoszcz, Nicolaus Copernicus University in Torun, Bydgoszcz, Poland.

4. Department of Otolaryngology and Laryngological Oncology, University of Medical Sciences, Poznan, Poland

5. Department of Oncologic Pathology and Prophylaxis, Poznan University of Medical Sciences & Greater Poland Cancer Center, Poznan, Poland

6. Department of Otorhinolaryngology, Head and Neck Surgery, Turku University Central Hospital and Turku University, Turku, Finland

7. Department of Tumor Pathology, Greater Poland Cancer Center, Poznan, Poland.


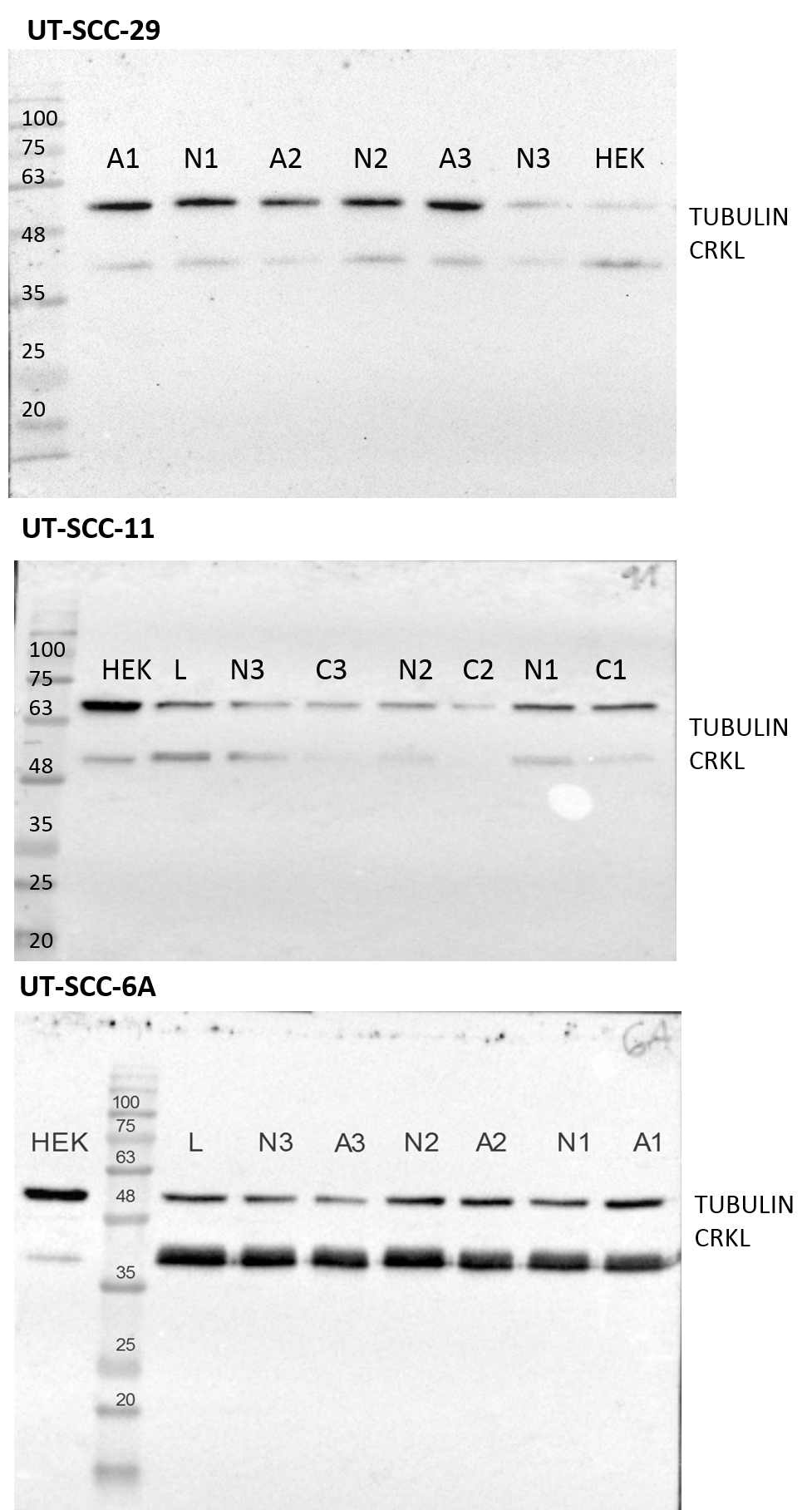


Supplementary Figure S1.

siRNA mediated *CRKL* knockdown in UT-SCC-29, UT-SCC-11 and UT-SCC-6A cell lines. Whole image of Western Blots.

A1, A2, A3, C1, C2, C3 siRNA duplexes targeting CRKL,

N1, N2, N3 negative siRNA as a transfection control,

L and HEK control without any siRNA and additional control.
